# Role of Macrophages and Plasminogen Activator Inhibitor-1 in Delayed Bone Repair Induced by Glucocorticoids in Mice

**DOI:** 10.3390/ijms23010478

**Published:** 2022-01-01

**Authors:** Kiyotaka Okada, Naoyuki Kawao, Daisho Nakai, Rei Wakabayashi, Yoshitaka Horiuchi, Katsumi Okumoto, Shinji Kurashimo, Yoshimasa Takafuji, Osamu Matsuo, Hiroshi Kaji

**Affiliations:** 1 Department of Arts and Sciences, Faculty of Medicine, Kindai University, Osaka 589-8511, Japan; kiyokada@med.kindai.ac.jp; 2 Department of Physiology and Regenerative Medicine, Faculty of Medicine, Kindai University, Osaka 589-8511, Japan; kawao@med.kindai.ac.jp (N.K.); 1610710131@edu.med.kindai.ac.jp (D.N.); 1610710087@edu.med.kindai.ac.jp (R.W.); takafuji@med.kindai.ac.jp (Y.T.); matsuo-o@med.kindai.ac.jp (O.M.); 3 Life Science Research Institute, Kindai University, Osaka 589-8511, Japan; yoshitaka.horiuchi@itp.kindai.ac.jp (Y.H.); katsmi.okumoto@itp.kindai.ac.jp (K.O.); kurashin@med.kindai.ac.jp (S.K.)

**Keywords:** glucocorticoids, bone repair, plasminogen activator inhibitor-1, macrophage

## Abstract

Glucocorticoids delay fracture healing and induce osteoporosis. However, the mechanisms by which glucocorticoids delay bone repair have yet to be clarified. Plasminogen activator inhibitor-1 (PAI-1) is the principal inhibitor of plasminogen activators and an adipocytokine that regulates metabolism. We herein investigated the roles of macrophages in glucocorticoid-induced delays in bone repair after femoral bone injury using PAI-1-deficient female mice intraperitoneally administered with dexamethasone (Dex). Dex significantly decreased the number of F4/80-positive macrophages at the damaged site two days after femoral bone injury. It also attenuated bone injury-induced decreases in the number of hematopoietic stem cells in bone marrow in wild-type and PAI-1-deficient mice. PAI-1 deficiency significantly weakened Dex-induced decreases in macrophage number and macrophage colony-stimulating factor (M-CSF) mRNA levels at the damaged site two days after bone injury. It also significantly ameliorated the Dex-induced inhibition of macrophage phagocytosis at the damaged site. In conclusion, we herein demonstrated that Dex decreased the number of macrophages at the damaged site during early bone repair after femoral bone injury partly through PAI-1 and M-CSF in mice.

## 1. Introduction

Glucocorticoids regulate numerous physiological processes [1,2]. They have been widely used to treat chronic inflammatory diseases due to their potent anti-inflammatory effects [3,4]. Osteoporosis is one of the well-known side effects of glucocorticoids; its pathogenesis mainly involves a decrease in osteoblastic bone formation, and treatment with high-dose glucocorticoids transiently increases bone resorption [5,6,7]. Previous findings suggested that excess glucocorticoids delay bone repair [5,6,7,8]; however, the underlying mechanisms remain unclear.

Three phases, the inflammatory, restoration, and bone remodeling phases, are included in the bone repair process after bone injury or fractures [9]. Neutrophils, macrophages, and lymphocytes accumulate at bone injury sites and participate in some immune reactions in the inflammatory phase. Exclusion of cell debris and damaged cells, the release of cytokines, or growth factors, vessel formation, and tissue repair are some of the actions of macrophages in bone repair [10]. Regarding the roles of macrophages in bone, extensive evidence suggests crucial roles for bone-specific macrophages, osteomacs, in osteoblastic bone formation and fracture healing [11,12,13,14]. In our research, we showed that the tissue fibrinolysis system was involved in the bone repair process after a bone defect in mice and was related to macrophages at the damaged site [15,16]. These findings indicated that macrophages play crucial roles in the bone repair process after a bone defect or fractures at the damaged site of bone injury.

The pathogenesis of delayed bone repair in the diabetic state has been attributed to various factors, including the impaired mobilization of mesenchymal and hematopoietic stem cells (HSCs), chondrogenesis, osteoblastic bone formation, and vessel formation [17,18,19]. We previously reported that plasminogen activator inhibitor-1 (PAI-1), an inhibitor of plasminogen activators, contributed to delayed bone repair and osteopenia induced by streptozotocin in female diabetic mice [20,21,22]. Moreover, we recently revealed that PAI-1 played a role in delayed bone repair induced by glucocorticoids in mice [8]. In that study, PAI-1, as an adipocytokine negatively affecting bone, appeared to affect osteoblast differentiation and apoptosis during the restoration phase of bone repair after a femoral bone defect in mice. These findings indicated that macrophages and PAI-1 are involved in glucocorticoid-induced delays in bone repair.

Therefore, we herein investigated the roles of macrophages in delayed bone repair after bone injury induced by glucocorticoids using dexamethasone (Dex), an active glucocorticoid. We also examined the impact of PAI-1 on the role of macrophages in bone repair using PAI-1-deficient (PAI-1 KO) mice and their wild-type (WT) counterparts treated with Dex.

## 2. Results

### 2.1. Effects of Dex and PAI-1 Deficiency on Macrophage Number after Femoral Bone Injury

**Figure 1:**
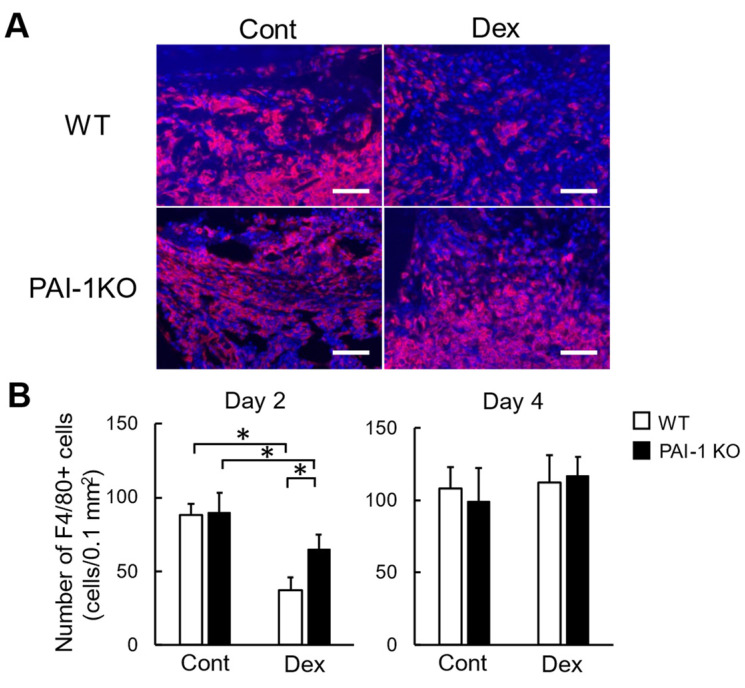
Effects of PAI-1 deficiency on Dex-induced decreases in macrophage number after femoral bone injury. (**A**) Microphotographs of F4/80-positive cells at damaged sites 2 days after femoral bone injury in control and Dex-treated WT and PAI-1 KO mice. Scale bars indicate 50 μm. (**B**) Number of F4/80-positive cells at damaged sites 2 and 4 days after femoral bone injury in control and Dex-treated WT and PAI-1 KO mice. Data represent the mean ± SEM: *n* = 5 mice in each group. * *p* < 0.05. Cont, control; DAPI, 4′,6-diamidino-2-phenylindole.

Consistent with our previous findings, the Dex treatment for three weeks elevated plasma PAI-1 levels without increasing fasting blood glucose levels in mice [8]. PAI-1 deficiency significantly lessened Dex-induced delays in bone repair in mice [8]. We then investigated the number of macrophages at the damaged site after femoral bone injury using the same glucocorticoid-treated mouse model. The Dex treatment significantly decreased the number of F4/80-positive cells at the damaged site two days after femoral bone injury in mice, while PAI-1 deficiency significantly lessened this reduction (Figure 1A,B). However, PAI-1 deficiency and the Dex treatment did not affect the number of F4/80-positive cells four days after bone injury in mice (Figure 1B).

### 2.2. Effects of Dex on the Number of HSCs in Bone Marrow after Femoral Bone Injury

**Figure 2:**
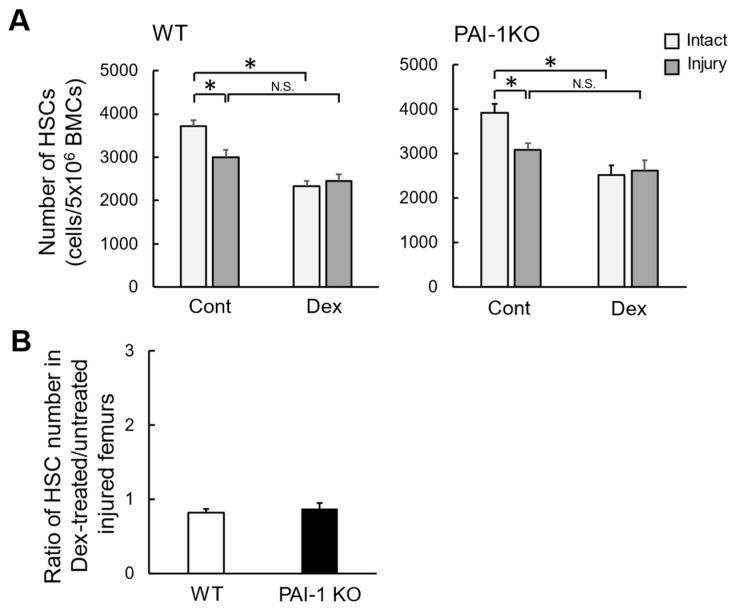
HSCs in bone marrow from damaged femurs after femoral bone injury. (**A**) The numbers of CD34^−^, c-Kit^+^, Sca-1^+^, and Lin^−^ cells (HSCs) harvested from the bone marrow of the contralateral intact (intact) and damaged (injury) femurs 2 days after femoral bone injury in WT (left panel) and PAI-1 KO (right panel) mice, as assessed by flow cytometry. Data represent the means ± SEM of 5 experiments (10 mice) in each group. * *p* < 0.05. BMC, bone marrow cell; Cont, control. N.S., not significant (**B**) Ratio of the bone marrow HSC number in Dex-treated/untreated WT and PAI-1 KO mice 2 days after femoral bone injury assessed by flow cytometry. Data represent the mean ± SEM of 5 mice.

Our previous study indicated that femoral bone injury induces a decrease in bone marrow HSC number in mice [23], which might partly influence macrophage number at the damaged site. We therefore examined the effects of Dex on bone marrow HSC number after femoral bone injury in mice. The prevalence of HSCs in bone marrow from damaged and contralateral intact femurs after femoral bone injury in WT and PAI-1 KO mice was evaluated using a flow cytometric analysis. HSCs were defined as cells that were CD34^−^, c-Kit^+^, Sca-1^+^, and Lin^−^. As shown in Figure 2, the number of HSCs harvested from the bone marrow of damaged femurs was significantly lower than that from the bone marrow of contralateral intact femurs two days after femoral bone injury in WT mice. The Dex treatment lessened the decrease in the number of HSCs induced by bone injury in WT mice; however, in comparisons with the control, the Dex treatment significantly reduced the number of HSCs in intact femurs (Figure 2A). Similar results were obtained for PAI-1 KO mice (Figure 2A). PAI-1 deficiency did not affect the Dex-induced reduction in the ratio of HSC numbers in the bone marrow of damaged femurs two days after femoral bone injury (Figure 2B). 

### 2.3. Effects of Dex on Macrophage-Related Gene Expression at the Damaged Site after Bone Injury

**Figure 3:**
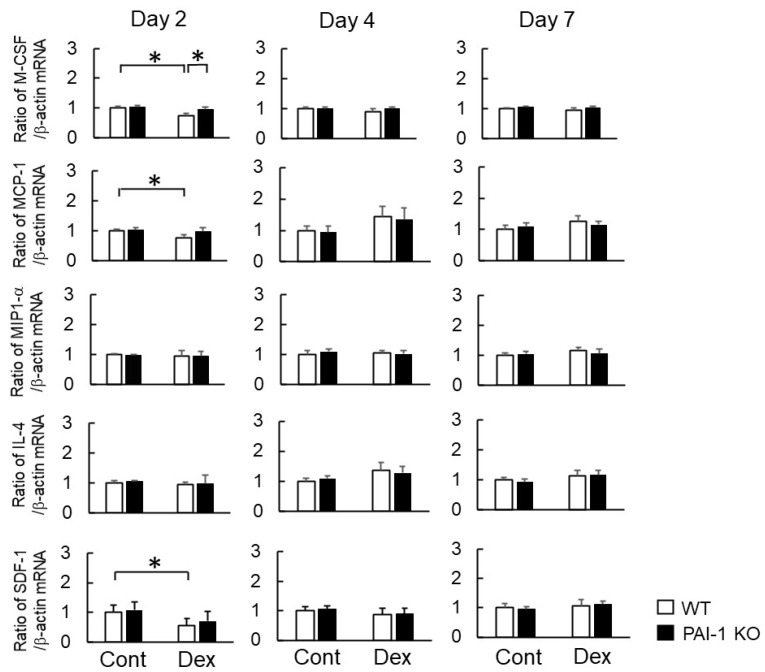
Effects of Dex and PAI-1 deficiency on the expression of macrophage-related factors at the damaged site after femoral bone injury. A real-time PCR analysis of *M-CSF*, *MCP-1*, *MIP-1α*, *IL-4*, *SDF-1*, and *β-actin* mRNA at damaged sites 2, 4, and 7 days after femoral bone injury in WT and PAI-1 KO mice. Data are expressed relative to *β-actin* mRNA values. Data represent the mean ± SEM: *n* = 5. * *p* < 0.05.

Macrophages secrete various cytokines and regulators into the microenvironment during inflammation and tissue repair. *Tumor necrosis factor-α (TNF-α)*, *interleukin (IL)-1β*, *inducible nitric oxide synthase (iNOS)*, and *IL-6* are M1 macrophage-synthesized factors, while *IL-10*, *CD206*, *arginase 1*, and *transforming growth factor-β (TGF-β)* are M2 macrophage-synthesized factors [10]. We examined the expression of macrophage-related genes in bone tissues at the damaged site two, four, and seven days after femoral bone injury in WT and PAI-1 KO mice. The Dex treatment significantly decreased the mRNA levels of *macrophage colony-stimulating factor (M-CSF)*, *monocyte chemoattractant protein (MCP)-1*, and *stromal cell-derived factor-1 (SDF-1)*, but not *macrophage inflammatory protein (MIP)-1α* or *IL-4*, at the damaged site two days after bone injury in WT mice (Figure 3). PAI-1 deficiency significantly lessened Dex-induced decreases in *M-CSF* mRNA levels at the damaged site (Figure 3), but did not affect the mRNA levels of *MCP-1*, *MIP-1α*, *IL-4*, or *SDF-1* in bone tissues at the damaged site two, four, and seven days after femoral bone injury (Figure 3). 

**Figure 4:**
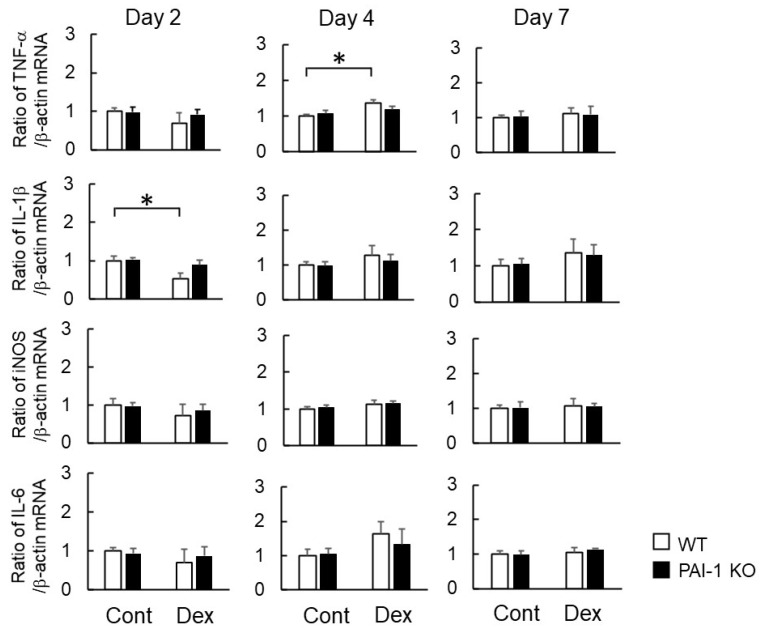
Effects of Dex and PAI-1 deficiency on the expression of M1 macrophage-producing factors at damaged sites after femoral bone injury. A real-time PCR analysis of the mRNA levels of *TNF-α*, *IL-1β*, *iNOS*, *IL-6*, and *β-actin* at damaged sites 2, 4, and 7 days after femoral bone injury in WT and PAI-1 KO mice. Data are expressed relative to *β-actin* mRNA values. Data represent the mean ± SEM: *n* = 5. * *p* < 0.05.

**Figure 5:**
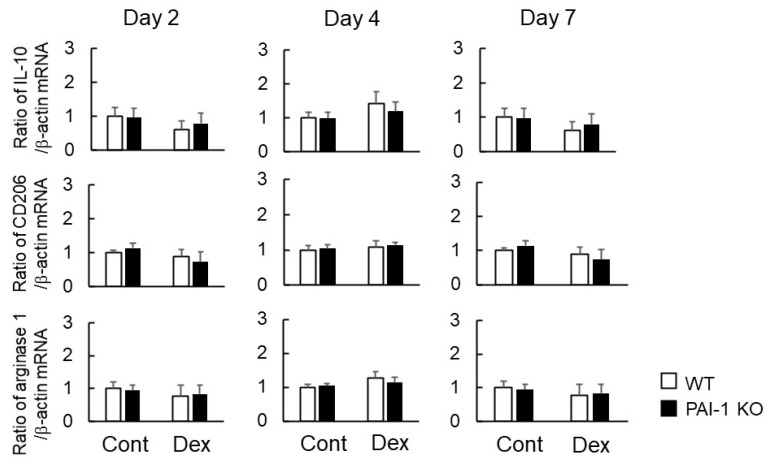
Effects of Dex and PAI-1 deficiency on the expression of M2 macrophage-producing factors at damaged sites after femoral bone injury. A real-time PCR analysis of the mRNA levels of *IL-10*, *CD206*, *arginase 1*, and *β-actin* at damaged sites 2, 4, and 7 days after femoral bone injury in WT and PAI-1 KO mice. Data are expressed relative to *β-actin* mRNA values. Data represent the mean ± SEM: *n* = 5.

Regarding the factors related to the phenotypes of M1 and M2 macrophages, the Dex treatment significantly increased *TNF-α* mRNA levels at the damaged site four days after bone injury in WT mice, but significantly decreased *IL-1β* mRNA levels at the damaged site two days after bone injury. PAI-1 deficiency appeared to weaken Dex-induced increases in *TNF-α* mRNA levels and decreases in *IL-1β* mRNA levels at the damaged site (Figure 4). Neither PAI-1 deficiency nor Dex affected the mRNA levels of *iNOS*, *IL-6*, *IL-10*, *CD206*, or *arginase 1* in bone tissues at the damaged site two, four, or seven days after femoral bone injury (Figure 4 and Figure 5).

### 2.4. Effects of Dex and PAI-1 Deficiency on Factors Produced from Macrophages Derived from the Bone Marrow of Damaged Femurs

**Figure 6:**
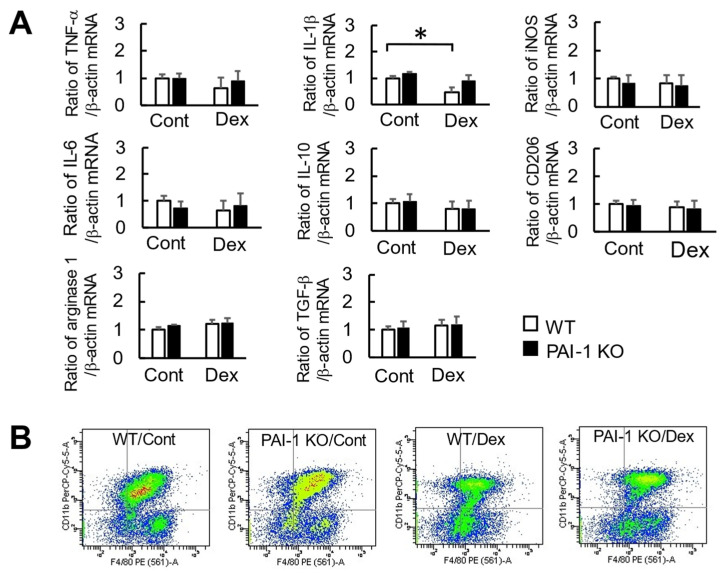
Effects of Dex and PAI-1 deficiency on the expression of macrophage-related factors in macrophages derived from the bone marrow of damaged femurs. (**A**) A real-time PCR analysis of *TNF-α*, *IL-1β*, *iNOS*, *IL-6*, *IL-10*, *CD206*, *arginase 1*, *TGF-β*, and *β-actin* mRNA in F4/80 and CD11b double-positive cells derived from the bone marrow of damaged femurs 2 days after bone injury in mice using fluorescence-activated cell sorting. Data are expressed relative to *0* mRNA values. Data represent the mean ± SEM of 5 mice in each group. * *p* < 0.05. (**B**) Flow cutometric analysis was used to identify F4/80 and CD11b double-positive cells from damaged femurs 2 days after bone injury.

To examine the effects of glucocorticoids and PAI-1 deficiency on the factors produced from macrophages, we examined the expression of macrophage-related factors in F4/80 and CD11b double-positive cells (putative macrophages) derived from the bone marrow of damaged femurs in mice two days after bone injury using fluorescence-activated cell sorting. Although the Dex treatment significantly decreased *IL-1β* mRNA levels in F4/80 and CD11b double-positive cells from the bone marrow of damaged femurs in WT mice, PAI-1 deficiency appeared to lessen Dex-induced reductions in IL-1β mRNA levels (Figure 6A). The Dex treatment did not affect the mRNA levels of *TNF-α*, *iNOS*, *IL-6*, *IL-10*, *CD206*, or *arginase 1* in F4/80 and CD11b double-positive cells from the bone marrow of damaged femurs in WT and PAI-1 KO mice (Figure 6A). FACS data of F4/80 and CD11b double positive cells are shown in Figure 6B. F4/80 and CD11b double positive cells were 45.8 ± 3.5% in whole bone marrow cells (Figure 6B).

### 2.5. Effects of Dex and PAI-1 Deficiency on the Phagocytosis of Macrophages at Damaged Femurs

**Figure 7:**
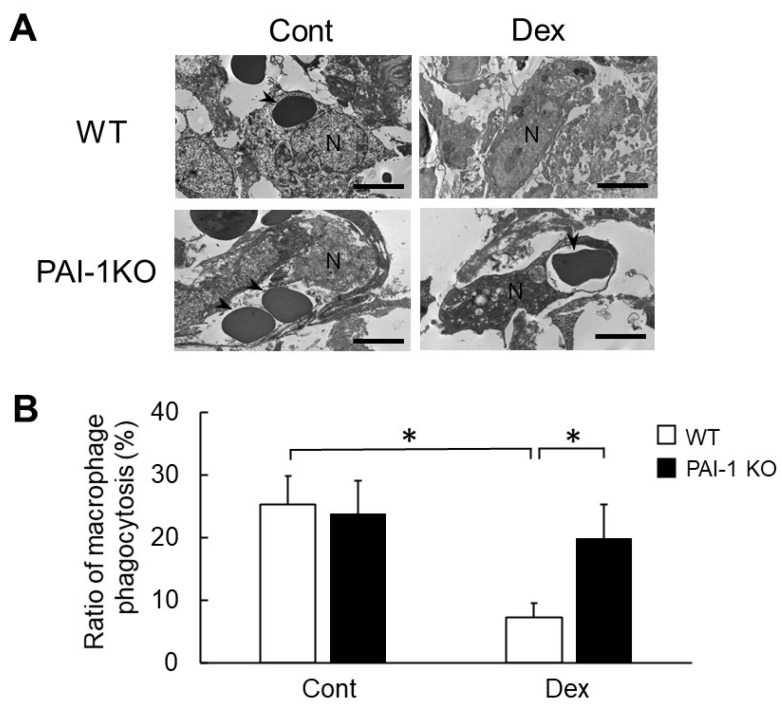
Effects of Dex and PAI-1 deficiency on macrophage phagocytosis after femoral bone injury. (**A**) Transmission electron microscopic photographs of macrophages in WT and PAI-1 KO mice treated with or without Dex at the damaged site 2 days after femoral bone injury. Results represent experiments performed on 4 mice in each group. Arrowheads indicate erythrocytes in macrophages. Scale bars indicate 5 μm. N, nucleus. (**B**) The activity of macrophage phagocytosis was calculated as the number of phagocytosing macrophages divided by the number of subject macrophages (ratio of macrophage phagocytosis) at the damaged site 2 days after femoral bone injury, assessed by transmission electron microscopy. Data represent the mean ± SEM of 4 mice in each group. * *p* < 0.05.

We then investigated the phagocytosis of macrophages at the damaged site two days after femoral bone injury using transmission electron microscopy in WT and PAI-1 KO mice. As shown in Figure 7A, recruited macrophages with well-extended pseudopodia were observed at the damaged site in WT and PAI-1 KO mice. These macrophages engulfed erythrocytes or cellular debris at the damaged site in WT and PAI-1 KO mice. Although the Dex treatment significantly reduced the ratio of macrophage phagocytosis at the damaged site in WT mice, PAI-1 deficiency significantly weakened the Dex-induced decrease in the ratio of macrophage phagocytosis in mice (Figure 7A,B).

## 3. Discussion

In the present study, we showed that Dex suppressed the number and phagocytosis of macrophages as well as the expression of *M-CSF*, *MCP-1*, *IL-1β*, and *SDF-1* at the damaged site two days after femoral bone injury during bone repair in mice. Moreover, PAI-1 deficiency attenuated Dex-induced decreases in the number and phagocytosis of macrophages as well as M-CSF expression at bone tissues in mice.

Previous studies suggested that glucocorticoids influence macrophage functions under pathophysiological conditions. They were shown to efficiently inhibit the tissue repair process by down-regulating pro-inflammatory mediators from macrophages and monocytes [24], but induced aberrant macrophage immune functions and apoptosis [25]. Glucocorticoids also inhibited macrophage differentiation towards a pro-inflammatory phenotype upon wounding, without affecting their migration [26]. In the present study, Dex suppressed the number of macrophages at the damaged site two days after femoral bone injury during bone repair in mice, which is consistent with previous findings showing the regulation of tissue repair and immune responses by glucocorticoids [23,24,25]. Previous studies proposed crucial roles for the bone’s resident macrophage population, osteomacs, in bone formation and bone repair after injury [11,12,13,14]. Moreover, recent evidence showed the functional involvement of osteomacs in bone repair or regeneration. Macrophage-lineage tartrate-resistant acid phosphatase-positive cells participate in periosteal osteogenesis and regeneration through the recruitment of periosteum-derived cells in mice [27]. Macrophage-secreting low-density lipoprotein receptor-related protein 1 or macrophage-expressed G-protein-coupled receptor-interacting protein 1 was shown to play a role in the fracture repair process in mice [28,29]. Qiao et al. reported that the sequential activation of heterogenous macrophage phenotypes, such as M1 and M2 macrophages, was necessary for the bone regeneration process in rats [30]. These findings suggest that macrophages are key players in the bone repair process, such as the inflammatory, restoration, and remodeling phases. Taken together, the present results indicate that glucocorticoids delayed bone repair after bone injury by affecting macrophages during the bone repair process.

Macrophages and neutrophils are important in tissue repair, particularly in the early phase [9]. On the other hand, a change in neutrophil accumulation was not observed at the damaged site after bone injury during the bone repair process, which was delayed by the diabetic state in our mouse study [31]. Moreover, we previously revealed that femoral bone injury induced a decrease in HSC numbers in the bone marrow during the bone repair process in mice [23], which is consistent with the present results. These findings suggest that the mobilization and recruitment of HSCs to damaged sites for the acceleration of bone repair after bone injury reduced HSC numbers in the bone marrow. We also previously indicated that HSC population changes by bone injury were blunted by the diabetic state in mice [31]. The impaired mobilization of stem cells in the bone marrow was suggested to be induced by glucocorticoids; however, this phenomenon may be related to a tissue repair reaction, inflammation, and vessel formation [32,33,34,35]. In the present study, macrophages and osteoclasts were differentiated from transplanted HSCs in bone marrow during the bone repair process after a femoral bone defect in mice [24]. Based on these findings, we speculate that glucocorticoids inhibit the mobilization and migration of bone marrow HSCs, leading to the impaired accumulation of macrophages at damaged sites during the bone repair process in mice. Alternatively, Dex treatment reduced HSC number in both intact and injured femurs in our data, suggesting that Dex might exert much more profound effects on HSC number than bone injury. Dex might therefore mask the effect of bone injury on the HSC number, instead of weakening it.

PAI-1 has been implicated in numerous inflammatory, metabolic, and endocrinological disorders as well as diabetes or estrogen deficiency-induced osteopenia [36]. We previously revealed that PAI-1 contributed to the diabetic state, osteopenia, and muscle wasting induced by a continuous pellet treatment with 1.5 mg of corticosterone, a glucocorticoid, in mice [37,38]. Moreover, we recently reported that PAI-1 deficiency lessened Dex-induced delays in bone repair after femoral bone injury in mice, indicating a role for PAI-1 in glucocorticoid-induced delays in bone repair after bone injury in mice. In the present study, PAI-1 deficiency significantly attenuated Dex-induced decreases in the number and phagocytosis of macrophages at the damaged site two days after femoral bone injury in mice, indicating that PAI-1 is involved in the glucocorticoid-mediated suppression of the number of macrophages and phagocytosis at the injured site during bone repair. Since PAI-1 deficiency did not affect Dex-induced decreases in the number of HSCs in bone marrow during bone repair in mice, the effects of PAI-1 on macrophage number at the bone injury site may not be due to its effects on the mobilization of HSCs from bone marrow to macrophages at the bone injury site. Our findings also suggested that the effects of glucocorticoids on osteoporosis were due to elevated serum PAI-1 levels secreted from adipose tissues, but not local PAI-1 production by bone tissues in mice; however, hepatic tissue appeared to be more important as the secretory organ for circulating PAI-1 in type 1 diabetic bone loss in female mice [21,37]. Excess glucocorticoids may elevate circulating PAI-1 levels, partly through an increase in the secretion of PAI-1 from adipose tissues resulting in delayed bone repair after bone injury in mice, because plasma levels of PAI-1, as well as its expression in adipose tissues and muscles, but not bone or the liver, were increased by Dex in mice.

M-CSF and IL-4 are important factors for macrophage differentiation and proliferation, respectively [11,39]. MCP-1 and MIP-1*α* are crucial for the accumulation of macrophages [39]. Macrophages are classified into two subtypes, M1 and M2. M1 macrophages are related to the inflammation process in tissue repair, and alternatively activated M2 macrophages participate in tissue regeneration [10,39]. However, inflammatory macrophages were recently proposed to participate in the bone repair process [9,30]. In the present study, Dex significantly decreased the expression of *M-CSF* in bone tissues at the damaged site two days after bone injury in mice. These results suggest that *M-CSF* is involved in glucocorticoid-induced decreases in macrophage accumulation at the damaged site after femoral bone injury, possibly through a decrease in the differentiation of macrophage precursor cells into macrophages. On the other hand, Dex significantly suppressed the expression of *MCP-1*, *IL-1β*, and *SDF-1* in bone tissues at the damaged site two days after bone injury in mice in the present study, but significantly up-regulated *TNF-α* expression at the damaged site four days after bone injury. Since bone tissues include various cell types other than macrophages, and the expression of cytokines may not be caused by macrophage-derived cells, we examined the expression of M1 and M2 macrophage-related factors in F4/80 and CD11b double-positive cells (putative macrophages) in the bone marrow of damaged bone two days after bone injury in mice. We found that Dex significantly decreased the expression of *IL-1β* in macrophages at damaged bone marrow two days after bone injury in mice. These results suggest that glucocorticoids modulate the production of factors from macrophages at the damaged site in the inflammation phase during bone repair after bone injury in mice.

The present results revealed that PAI-1 deficiency significantly blunted the number of macrophages and *M-CSF* expression decreased by Dex at the damaged site two days after bone injury in mice. These results suggest that glucocorticoids delay bone repair by decreasing macrophage number partly through PAI-1 and suppressing M-CSF production from damaged bone tissues in mice. However, further studies using bone-specific M-CSF-deficient mice are needed to clarify the involvement of M-CSF in the regulation of the bone repair process by endocrine and metabolic disorders, including excess glucocorticoids. On the other hand, PAI-1 deficiency slightly reversed Dex-induced decreases in the expression of *MCP-1* and *IL-1β* at the damaged site two days after bone injury in mice. Therefore, we cannot rule out the possibility that other factors, such as *MCP-1* and *IL-1β*, are involved in glucocorticoid-induced delays in bone repair by decreasing the accumulation of macrophages partly through PAI-1 in mice.

MacCauley et al. recently reported that the percentage of M1 macrophages is elevated seven days after femoral fractures in mice [40], although the number of macrophages and expressions of macrophage-related genes other than *TNF-α* at the damaged site were not altered four days after femoral injury in our study. These differences might be due to the differences of time course for bone repair after bone injury between fracture model and pinhole bone defect method in mice, since bone repair after fracture slowly progresses in general, compared to that after pinhole bone defect. Otherwise, the differences in assay sensitivity of immune stain or the bone marrow samples from the damaged site might influence the differences in the results from different methods.

In conclusion, this is the first study to demonstrate that Dex decreased the number and phagocytosis of macrophages at the site of bone injury during early bone repair through PAI-1 in mice. Macrophages and PAI-1 may be targets for the treatment of delayed bone repair as a side effect of glucocorticoids.

## 4. Materials and Methods

### 4.1. Animal Experiments

Female PAI-1 KO mice and their WT counterparts, initially provided by D. Collen (University of Leuven, Leuven, Belgium), each weighing between 18 and 25 g, aged 9 weeks old, and with a mixed *C57BL/6J* (81.25%) and *129/SvJ* (18.75%) background, were used. Nine-week-old female WT and PAI-1 KO mice were intraperitoneally administered 2 mg/kg Dex (Wako, Tokyo, Japan) and normal saline once a day for three weeks [8]. Animals were maintained in metabolic cages under a 12-h light/dark cycle and received food and water ad libitum.

### 4.2. Murine Bone Injury Model

Bone injury was induced in mice according to a previously described method [8]. Briefly, under anesthesia induced by 2% isoflurane, a 5-mm-long longitudinal incision was made in the anterior skin over the mid-femur of the right leg. After splitting the muscle, the surface of the femoral bone was exposed. Thereafter, a hole was made using a drill with a diameter of 0.9 mm. The hole was irrigated with saline to prevent thermal necrosis of the margins. Incised skin was then sutured in a sterile manner, and anesthesia was discontinued.

### 4.3. Histological Analysis

Mice were anesthetized using 2% isoflurane on day zero, two, four, or seven after femoral bone injury. Femurs were removed, fixed in 4% paraformaldehyde, demineralized in 22.5% formic acid and 340 mM sodium citrate solution for 24 h, and embedded in paraffin. Immunostaining was performed as previously described [15]. To evaluate the number of osteoblastic cells at the bone surface, sections were incubated with an anti-F4/80 antibody (AbD Serotec, Raleigh, NC, USA) at a dilution of 1:20, followed by an incubation with an appropriate horseradish peroxidase-conjugated secondary antibody. Positive signals were visualized using the tyramide signal amplification system (PerkinElmer, Waltham, MS, USA), and sections were counterstained with 4′,6-diamidino-2-phenylindole and photographed using an All-in-One Fluorescence Microscope (KEYENCE, Osaka, Japan).

### 4.4. Flow Cytometric Analysis

Bone marrow cells were obtained from mice as previously described [23]. Hank’s balanced salt solution buffer with 2% fetal bovine serum (FBS) was used to flush bone marrow cells from the harvested femurs of two mice. Bone marrow cells were added to an equivalent volume of Ficoll-Paque PLUS (GE Healthcare Bio-Sciences, Uppsala, Sweden) and harvested by centrifugation at 630× *g* at 4 °C for 15 min. Cells were resuspended in phosphate-buffered saline supplemented with 3% FBS and then analyzed with a FACS Aria II cell sorter (BD Biosciences, San Jose, CA, USA) HSCs were identified in bone marrow cell populations with Alexa 700 conjugated anti-CD34, BV711-conjugated anti-c-kit, and PE-Cy7-conjugated anti-Sca-1 antibodies and the peridinin-chlorophyll protein complex-Cy5.5-conjugated anti-lineage antibodies cocktail (BD Biosciences, San Jose, CA, USA). The numbers of HSCs harvested from the bone marrow of the contralateral intact and damaged femurs on day two after femoral bone injury were enumerated by flow cytometry.

### 4.5. Quantitative Real-Time Polymerase Chain Reaction (PCR)

Total RNA was isolated from tissues and cells using an RNeasy Mini Kit (Qiagen, Hilden, Germany). The incorporation of SYBR Green into double-stranded DNA was assessed by quantitative real-time PCR using an ABI StepOne Real-Time PCR System (Applied Biosystems, Carlsbad, CA, USA), as previously described [8]. The PCR primers used are listed in Table 1. The mRNA levels of the target genes were normalized with β-actin mRNA levels.

### 4.6. Isolation of F4/80 and CD11b Double-Positive Cells from the Femur

Bone marrow cells were flushed out into Dulbecco’s modified Eagle medium (DMEM) with 1% penicillin streptomycin. Cells were labeled at 4 °C for 30 min with the optimal dilution of a phycoerythrin-conjugated anti-F4/80 antibody (AbD Serotec, Raleigh, NC, USA) and peridinin-chlorophyll protein-Cy5.5-conjugated anti-CD11b antibody (BD Biosciences, San Jose, CA, USA). After the lysis of erythrocytes, F4/80 and CD11b double-positive cells (5.0 × 10^5^) were isolated with an FACS Aria II cell sorter (BD Biosciences, San Jose, CA, USA) and analyzed by real-time PCR, as previously described [15].

### 4.7. Transmission Electron Microscopy

A transmission electron microscopy analysis was performed as previously described [16]. Briefly, mice were transcardially perfused with physiological saline, and then with 2.5% glutaraldehyde in phosphate buffer (pH 7.4), on day two after femoral bone injury. Femurs were removed, demineralized in 22.5% formic acid and 340 mM sodium citrate solution, and postfixed in the same fixative at 4 °C overnight. After fixation in 1% buffered osmium tetroxide and prestaining with 0.5% uranyl acetate, small pieces of the femurs were embedded in epoxy resin, and 70-nm-thick sections were obtained from the damaged site. Ultrathin sections were stained with 3% uranyl acetate at room temperature for 20 min. Stained sections were photographed with an electron microscope (HT-7700; Hitachi High-Technologies Co., Tokyo, Japan) at an accelerating voltage of 100 kV. The activity of macrophage phagocytosis at the damaged site of the femur was quantitatively analyzed as previously described [16]. Briefly, ≥25 macrophages at the damaged site of the femur were photographed in each mouse, and the number of phagocytosing macrophages with erythrocytes or cellular debris >2 μm in diameter was quantified in a blinded manner. The ratio of phagocytosing macrophages to subject macrophages was calculated in each mouse for the assessment of phagocytosis activity.

### 4.8. Statistical Analysis

Data are presented as means ± standard errors of the mean (SEM). Statistical analyses were performed by the Mann–Whitney U test for comparisons of two groups. A two-way ANOVA followed by Tukey’s test was conducted for multiple comparisons. Differences of *p* < 0.05 were considered to be significant. All statistical analyses were performed using StatView v5.0 software (Statistical Analysis System (SAS) Institute Inc.; Cary, NC, USA).

## Figures and Tables

**Table 1:** Gene names and primer sequences used in this study.

Gene Name and Symbol	Forward Primer	Reverse Primer
*Macrophage colony-stimulating factor (MCSF)*	GACTTCATGCCAGATTGCC	GGTGGCTTTAGGGTACAGG
*Monocyte chemoattractant protein-1 (MCP-1)*	CCACTCACCTGCTGCTACTCA	TGGTGATCCTCTTGTAGCTCTCC
*Macrophage inflammatory protein-1α (MIP-1α)*	CCTCTGTCACCTGCTCAACA	GATGAATTGGCGTGGAATCT
*Interleukin-4 (IL-4)*	ACAGGAGAAGGGACGCCAT	GAAGCCCTACAGACGAGCTCA
*Interleukin-6 (IL-6)*	GTTCTCTGGGAAATCGTGGA	GGAAATTCGGGGTAGGAAGGA
*Tumor necrosis factor-α (TNF-α)*	CCCAGACCCTCACACTCAGATC	GCCACTCCAGCTGCTCCTC
*Interleukin-1β (IL-1β)*	GGTCAAAGGTTTGGAAGCAG	TGTGAAATGCCACCTTTTGA
*Inducible nitric oxide synthase (iNOS)*	TTTGCTTCCATGCTAATGCGAAAG	GCTCTGTTGAGGTCTAAAGGCTCCG
*Interleukin-10 (IL-10)*	GCTCTTACTGACTGGCATGAG	CGCAGCTCTAGGAGCATGTG
*CD206*	TTTGGAATCAAGGGCACAGAG	TGCTCCACAATCCCGAACC
*Arginase1*	CTCCAAGCCAAAGTCCTTAGAG	AGGAGCTGTCATTAGGGACATC
*Transforming growth factor-β_1_ (TGF-β_1_)*	GCAACAATTCCTGGCGTTACC	CGCTGAATCGAAAGCCCTGTA
*Stromal-derived factor-1 (SDF-1)*	CTGTGCCCTTACGATTGTTG	TCAGCCTTCCTCGGGGGTCT
*β-actin*	TACCACAGGCATTGTGATGG	TTTGATGTCACGCACGATTT

## Data Availability

The data presented in this study are available within the article text, tables, and figures.

## References

[1] Hofbauer L.C., Rauner M. (2009). Minireview: Live and let die: Molecular effects of glucocorticoids on bone cells. Mol Endocrinol..

[2] McInnes I.B., Schett G. (2011). The pathogenesis of rheumatoid arthritis. N. Engl. J. Med..

[3] Rhen T., Cidlowski J.A. (2005). Antiinflammatory action of glucocorticoids—New mechanisms for old drugs. N. Engl. J. Med..

[4] Strehl C., Buttgereit F. (2013). Optimized glucocorticoid therapy: Teaching old drugs new tricks. Mol. Cell. Endocrinol..

[5] Hachemi Y., Rapp A.E., Picke A.K., Weidinger G., Ignatius A., Tuckermann J. (2018). Molecular mechanisms of glucocorticoids on skeletal and bone regeneration after fracture. J. Mol. Endocrinol..

[6] Henneicke H., Gasparini S.J., Brennan-Speranza T.C., Zhou H., Seibel M.J. (2014). Glucocorticoids and bone: Local effects and systemic implications. Trends Endocrinol. Metab..

[7] Moutsatsou P., Kassi E., Papavassiliou A.G. (2012). Glucocorticoid receptor signaling in bone cells. Trends Mol. Med..

[8] Okada K., Okamoto T., Okumoto K., Takafuji Y., Ishida M., Kawao N., Matsuo O., Kaji H. (2020). PAI-1 is involved in delayed bone repair induced by glucocorticoids in mice. Bone.

[9] Claes L., Recknagel S., Ignatius A. (2012). Fracture healing under healthy and inflammatory conditions. Nat. Rev. Rheumatol..

[10] Gordon S., Martinez F.O. (2010). Alternative activation of macrophages: Mechanism and functions. Immunity.

[11] Alexander K.A., Chang M.K., Maylin E.R., Kohler T., Muller R., Wu A.C., Van Rooijen N., Sweet M.J., Hume D.A., Raggatt L.J. (2011). Osteal macrophages promote in vivo intramembranous bone healing in a mouse tibial injury model. J. Bone Miner. Res..

[12] Sinder B.P., Pettit A.R., McCauley L.K. (2015). Macrophages: Their emerging roles in bone. J Bone Miner. Res..

[13] Vi L., Baht G.S., Whetstone H., Ng A., Wei Q., Poon R., Mylvaganam S., Grynpas M., Alman B.A. (2015). Macrophages promote osteoblastic differentiation in-vivo: Implications in fracture repair and bone homeostasis. J. Bone Miner. Res..

[14] Miron R.J., Bosshardt D.D. (2016). OsteoMacs: Key players around bone biomaterials. Biomaterials.

[15] Kawao N., Tamura Y., Okumoto K., Yano M., Okada K., Matsuo O., Kaji H. (2013). Plasminogen plays a crucial role in bone repair. J. Bone Miner. Res..

[16] Kawao N., Tamura Y., Horiuchi Y., Okumoto K., Yano M., Okada K., Matsuo O., Kaji H. (2015). The tissue fibrinolytic system contributes to the induction of macrophage function and CCL3 during bone repair in mice. PLoS ONE.

[17] Retzepi M., Donos N. (2010). The effect of diabetes mellitus on osseous healing. Clin. Oral Implants Res..

[18] Shoji T., Koyama H., Morioka T., Tanaka S., Kizu A., Motoyama K., Mori K., Fukumoto S., Shioi A., Shimogaito N. (2006). Receptor for advanced glycation end products is involved in impaired angiogenic response in diabetes. Diabetes.

[19] Ko K.I., Coimbra L.S., Tian C., Alblowi J., Kayal R.A., Einhorn T.A., Gerstenfeld L.C., Pignolo R.J., Graves D.T. (2015). Diabetes reduces mesenchymal stem cells in fracture healing through a TNF-α-mediated mechanism. Diabetologia.

[20] Mao L., Kawao N., Tamura Y., Okumoto K., Okada K., Yano M., Matsuo O., Kaji H. (2014). Plasminogen activator inhibitor-1 is involved in impaired bone repair associated with diabetes in female mice. PLoS ONE.

[21] Tamura Y., Kawao N., Okada K., Yano M., Okumoto K., Matsuo O., Kaji H. (2013). Plasminogen activator inhibitor-1 is involved in streptozotocin-induced bone loss in female mice. Diabetes.

[22] Mao L., Tamura Y., Kawao N., Okada K., Yano M., Okumoto K., Kaji H. (2014). Influence of diabetic state and vitamin D deficiency on bone repair in female mice. Bone.

[23] Okada K., Kawao N., Yano M., Tamura Y., Kurashimo S., Okumoto K., Kojima K., Kaji H. (2016). Stromal cell-derived factor-1 mediates changes of bone marrow stem cells during the bone repair process. Am. J. Physiol. Endocrinol. Metab..

[24] Ehrchen J.M., Roth J., Barczyk-Kahlert K. (2019). Front more than suppression: Glucocorticoid action on monocytes and macrophages. Front. Immunol..

[25] Ai F., Zhao G., Lv W., Liu B., Lin J. (2020). Dexamethasone induces aberrant macrophage immune function and apoptosis. Oncol. Rep..

[26] Xie Y., Tolmeijer S., Oskam J.M., Tonkens T., Meijer A.H., Schaaf M.J.M. (2019). Glucocorticoids inhibit macrophage differentiation towards a pro-inflammatory phenotype upon wounding without affecting their migration. Dis. Models Mech..

[27] Gao B., Deng R., Chai Y., Chen H., Hu B., Wang X., Zhu S., Cao Y., Ni S., Wan M. (2019). Macrophage-lineage TRAP^+^ cells recruit periosteum-derived cells for periosteal osteogenesis and regeneration. J. Clin. Investig..

[28] Vi L., Baht G.S., Soderblom E.J., Whetstone H., Wei Q., Furman B., Puviindran V., Nadesan P., Foster M., Poon R. (2018). Macrophage cells secrete factors including LRP1 that orchestrate the rejuvenation of bone repair in mice. Nat. Commun..

[29] Zhao S.J., Liu H., Chen J., Qian D.F., Kong F.Q., Jie J., Yin G.Y., Li Q.Q., Fan J.J. (2020). Macrophage GIT1 contributes to bone regeneration by regulating inflammatory responses in an ERK/NRF2-dependent way. Bone Miner. Res..

[30] Qiao W., Xie H., Fang J., Shen J., Li W., Shen D., Wu J., Wu S., Liu X., Zheng Y. (2021). Sequential activation of heterogeneous macrophage phenotypes is essential for biomaterials-induced bone regeneration. Biomaterials.

[31] Shimoide T., Kawao N., Tamura Y., Okada K., Horiuchi Y., Okumoto K., Kurashimo S., Ishida M., Tatsumi K., Matsuo O. (2018). Role of macrophages and plasminogen activator inhibitor-1 in delayed bone repair in diabetic female mice. Endocrinology.

[32] Albiero M., Poncina N., Ciciliot S., Cappellari R., Menegazzo L., Ferraro F., Bolego C., Cignarella A., Avogaro A., Fadini G.P. (2015). Bone marrow macrophages contribute to diabetic stem cell mobilopathy by producing oncostatin M. Diabetes.

[33] Hazra S., Jarajapu Y.P., Stepps V., Caballero S., Thinschmidt J.S., Sautina L., Bengtsson N., Licalzi S., Dominguez J., Kern T.S. (2013). Long-term type 1 diabetes influences haematopoietic stem cells by reducing vascular repair potential and increasing inflammatory monocyte generation in a murine model. Diabetologia.

[34] Oikawa A., Siragusa M., Quaini F., Mangialardi G., Katare R.G., Caporali A., van Buul J.D., van Alphen F.P., Graiani G., Spinetti G. (2010). Diabetes mellitus induces bone marrow microangiopathy. Arterioscler. Thromb. Vasc. Biol..

[35] Fadini G.P., Albiero M., de Kreutzenberg S.V., Boscaro E., Cappellari R., Marescotti M., Poncina N., Agostini C., Avogaro A. (2013). Diabetes impairs stem cell and proangiogenic cell mobilization in humans. Diabetes Care.

[36] Kaji H. (2016). Adipose tissue-derived plasminogen activator inhibitor-I function and regulation. Comp. Physiol..

[37] Tamura Y., Kawao N., Yano M., Okada K., Okumoto K., Chiba Y., Matsuo O., Kaji. H. (2015). Role of plasminogen activator inhibitor-1 in glucocorticoid-induced diabetes and osteopenia in mice. Diabetes.

[38] Tamura Y., Kawao N., Shimoide T., Okada K., Matsuo O., Kaji H. (2018). Role of plasminogen activator inhibitor-1 in glucocorticoid-induced muscle change in mice. J. Bone Miner. Metab..

[39] Ono T., Takayanagi H. (2017). Osteoimmunology in bone fracture healing. Curr. Osteoporos. Rep..

[40] McCauley J., Bitsaktsis C., Cottrell J. (2020). Macrophage subtype and cytokine expression characterization during the acute inflammatory phase of mouse bone fracture repair. J. Orthop. Res..

